# Experiences of physiotherapists in managing pelvic floor disorders at public hospitals in uThukela district, South Africa

**DOI:** 10.4102/sajp.v82i1.2372

**Published:** 2026-09-30

**Authors:** Yashoda T. Naidoo, Joyful Msomi, Nombeko Mshunqane

**Affiliations:** 1 Department of Physiotherapy, Faculty of Health Sciences, University of KwaZulu-Natal, Durban, South Africa

**Keywords:** pelvic floor disorders, physiotherapy, qualitative study, undergraduate education, South Africa

## Abstract

**Background:**

Pelvic floor disorders (PFDs) are common among women in the uThukela district; however, many cases are not managed by physiotherapists in public healthcare settings. Limited emphasis on pelvic health during undergraduate physiotherapy training, combined with understaffing, inadequate resources and stigma associated with pelvic floor conditions, may contribute to this gap.

**Objectives:**

This study aimed to explore and describe the experiences of physiotherapists in managing patients with PFDs at public hospitals in the uThukela district, KwaZulu-Natal, South Africa.

**Method:**

A qualitative descriptive study was conducted with 12 physiotherapists from three district hospitals in the uThukela district, selected through purposive sampling. Data were collected using telephonic semi-structured interviews guided by a pre-piloted interview schedule. Interviews were audio-recorded, transcribed verbatim and analysed thematically using ATLAS.ti, following Braun and Clarke’s six-step approach. Trustworthiness was ensured through investigator triangulation, reflexivity, peer debriefing and maintenance of an audit trail.

**Results:**

Five themes emerged: undergraduate pelvic health training, barriers experienced by physiotherapists, patient-related barriers to accessing physiotherapy, current management approaches and strategies to improve management of PFDs. Most participants reported managing PFD cases infrequently, typically one patient every 6 months.

**Conclusion:**

Physiotherapists reported limited undergraduate training, inadequate resources, understaffing, poor referral pathways and patient-related barriers, which collectively hinder the management of PFDs in public hospitals. Strengthening pelvic health education, improving referral systems, increasing resource allocation and enhancing awareness among healthcare practitioners and patients may improve PFD management.

**Clinical Implications:**

This study identifies context-specific barriers, training gaps and limitations in managing pelvic floor disorders, informing education, delivery and referral improvements.

## Introduction

Pelvic floor disorders (PFDs) can be described as an impairment of the pelvic floor muscles, resulting in hypo- or hypertonicity of the pelvic floor (Nava et al. 2024). This can present as pelvic organ prolapse, stress and urge urinary incontinence and faecal incontinence (Nygaard et al. 2017). Women’s health has been a sub-discipline of physiotherapy since the early 19th century (Reddy & Frantz 2013) and has since expanded into men’s health. Physiotherapy plays a crucial role in the management and prevention of pelvic health conditions in both males and females (Boissonnault et al. 2020), through education and pelvic floor muscle training (Bernard et al. 2023). The prevalence of PFDs in South Africa is high (Moore 2021). It is expected to increase annually by 35% within the next 12 years (Alaniz et al. 2020). A study conducted in sub-Saharan Africa indicated that one in five women presents with urinary incontinence (Janse van Vuuren, Van Rensburg & Hanekom 2023); however, these statistics may be an underestimation due to under-reporting, as pelvic floor dysfunction is undertreated and under-investigated (Brandt 2025). The results of a South African study conducted in Ladysmith indicate a high prevalence of urinary incontinence among women in the town, with most of them not using pelvic floor exercises as a form of treatment (Madombwe 2010). As indicated above, the prevalence of PFDs is high; however, there is a lack of pelvic health management physiotherapy globally. Global research indicates the need for more trained professionals to deliver pelvic rehabilitative care (Nava et al. 2024).

In accordance with available research, there are many barriers that prevent physiotherapists from assessing for and treating PFDs. A study conducted in primary healthcare facilities in the Western Cape province, South Africa, indicates that 86.6% of healthcare professionals do not refer patients with pelvic conditions to physiotherapists (Janse van Vuuren et al. 2023), possibly due to a lack of education on the role and effectiveness of physiotherapy techniques in pelvic health (Reddy & Frantz 2013). Other factors that influence pelvic health patient care include the lack of confidence that physiotherapists have in their pelvic health knowledge (Reddy & Frantz 2013), a lack of PFD assessment and treatment protocols available (Reddy & Frantz 2013), religious restrictions that prevent physiotherapists from engaging in sensitive fields (Taak et al. 2024), the stigma attached to pelvic dysfunction may prevent patients from seeking help, the little attention that undergraduate physiotherapy programmes give to pelvic health (Brandt 2025), the lack of available postgraduate physiotherapy pelvic health training programmes (Brandt 2025), and patients lack the knowledge on PFD physiotherapy treatment (Mckay et al. 2019).

Although studies have delved into the prevalence of PFDs and physiotherapy management, none have investigated what physiotherapists are currently experiencing in the South African public health sector in pelvic health management. Hence, the aim of the study was to explore and describe experiences of physiotherapists in managing patients with PFDs at public hospitals in the uThukela district, South Africa.

## Research methods and design

### Research design

The study employed an exploratory descriptive qualitative design, using semi-structured interviews to explore the experiences of physiotherapists in managing PFDs at public hospitals in the uThukela district. A non-probability purposive sampling method was used to recruit community service and permanent physiotherapists working in the three public hospitals in the district. The study population consisted of all physiotherapists (*n* = 13) employed by Emmaus, Ladysmith and Estcourt hospitals. Twelve out of the 13 identified physiotherapists participated in the study.

An interview guide was developed based on the gaps identified in the research literature review and the study’s objectives. The interview guide was divided into five sections to address the study objectives: Section A: the demographic information of the participants, Section B: the barriers and facilitators to pelvic health management, Section C: undergraduate pelvic health training and current skills, Section D: changes needed to improve pelvic health management and Section E: screening and referrals of pelvic health patients. Probing questions were used to elicit further information from participants and expand on their answers.

A pilot study was conducted to ensure quality control of the study. Two physiotherapists working in the private sector were interviewed using the interview guide. The pilot study employed the same procedures as the main study. The data from the pilot were not included in the final data analysis. The outcome of the pilot study indicated that the consent form and instructions are clearly understood by the participant, the data collection procedure is manageable, 25 min – 35 min is enough time to conduct the interview, the questions in the interview guide are not misinterpreted, the open-ended questions allow the participants to expand on their answers, and extra probing questions were developed to obtain more information from participants.

### Data analysis

Interviews were transcribed verbatim by the author and analysed thematically using ATLAS.ti software. Data analysis followed Braun and Clarke’s six-step thematic analysis approach (Clarke & Braun 2013). The author first familiarised herself with the data through repeated reading of transcripts and review of interview recordings. Initial codes were then generated and grouped into categories. Similar codes were combined into themes and sub-themes, which were reviewed and refined to ensure they accurately represented the dataset. Themes were subsequently defined, named and supported with verbatim participant quotations. Data collection and analysis occurred concurrently, allowing the author to monitor data saturation throughout the study.

### Trustworthiness

Trustworthiness was established using Lincoln and Guba’s (1985) criteria of credibility, dependability, confirmability and transferability. Credibility was enhanced through investigator triangulation, prolonged engagement during interviews and peer review of the research process and findings. Dependability was ensured through reflexive practice and maintenance of an audit trail documenting methodological decisions and theme development. Confirmability was strengthened through ongoing peer debriefing with the research supervisors. Transferability was enhanced through purposive sampling and detailed descriptions of the study setting, participants and context.

### Ethical considerations

Ethical clearance to conduct this study was obtained from the Human and Social Sciences Research Ethics Committee of the University of KwaZulu-Natal and the KwaZulu-Natal Department of Health (No. HSSREC/00008677/2025 and KZ_202507_003).

A permission letter was sent for approval by the uThukela District Medical Director. Thereafter, gatekeeper permission letters were sent to and approved by each hospital chief executive officer. Once permission was granted from hospital management, each physiotherapy department was contacted to conduct the study. Informed consent was obtained from each participant by having them sign a consent form. The signed consent form was emailed to the author before the interview was conducted. Once consent was cleared, online interviews were scheduled, and each participant was emailed a link to the meeting. The 30 min – 45-min meetings were audio-recorded, and participants could withdraw at any time.

## Results

A total of 12 physiotherapists were interviewed. Table 1 summarises the demographic information of the participants. There were more females (*n* = 8) than males (*n* = 4). The ages and years of experience of the participants were categorised. Most physiotherapists are qualified from South African universities, and only one is qualified from a University in Tunisia.

**TABLE 1:** Summary statistics regarding demographic information (*N* = 12).

Participant characteristic	Category	Number of participants
Gender	Male	4
	Female	8
Age (years)	21–30	4
	31–40	4
	41–50	2
	51–60	2
Years of physiotherapy experience	0–10	5
	11–20	4
	21–30	3
CommServe or Permanent	Community service physiotherapist	4
	Permanent physiotherapist	8
Hospital distribution	Ladysmith Hospital	7
	Estcourt Hospital	4
	Emmaus Hospital	1
University attended	University of KwaZulu-Natal	9
	Sefako Makgatho University	2
	International university	1

## Discussion

This study explored physiotherapists’ experiences in managing PFDs, with findings organised into five key themes (Table 2).

**TABLE 2:** Summary of themes, sub-themes and verbatim quotes.

Themes	Sub-themes	Quotes
1. Undergraduate pelvic health training	1.1. Assessment skills 1.2. Treatment skills	1.1.1. ‘My undergraduate programme had very little exposure to pelvic health.’ (P1, male, permanent physiotherapist) 1.1.2. ‘We were not taught any pelvic floor exercises or treatment techniques.’ (P2, female, permanent physiotherapist) 1.1.3. ‘My undergraduate programme covered pelvic health vaguely.’ (P3, male, community service physiotherapist)
2. Physiotherapists’ barriers to managing PFD	2.1. Religious or cultural barriers 2.2. Gender barriers 2.3. Lack of knowledge 2.4. Lack of resources 2.5. Lack of referrals	2.1.1. ‘My religion does not allow me to treat males.’ (P4, female, permanent physiotherapist) 2.1.2. ‘I am not comfortable to expose a female patient.’ (P7, male, permanent physiotherapist) 2.1.3. ‘I am not confident in my pelvic health knowledge due to the lack of training.’ (P3, male, community service physiotherapist) 2.1.4. ‘I am not comfortable to expose a female patient.’ (P7, male, permanent physiotherapist) 2.1.5. ‘We do not have private treatment areas.’ (P2, female, permanent physiotherapist) 2.1.6. ‘We receive few pelvic patient referrals from doctors within the hospital.’ (P8, female, permanent physiotherapist)
3. Patients’ barriers to accessing physiotherapy	3.1. Stigma around pelvic health 3.2. Unaware of the physiotherapist’s role in pelvic health 3.3. Gender barriers 3.4. Cultural barriers 3.5. Lack access to healthcare	3.1.1. ‘There is a stigma attached to pelvic health; hence they may not approach us for help.’ (P11, male, permanent physiotherapist) 3.1.2. ‘They don’t know that physiotherapists can treat their pelvic symptoms.’ (P10, female, community service physiotherapist) 3.1.3. ‘Women are often taught that urinary incontinence is normal.’ (P8, female, permanent physiotherapist) 3.1.4. ‘Men are shy to talk about their symptoms.’ (P12, female, permanent physiotherapist) 3.1.5. ‘Many patients cannot afford the transport to get to the hospital.’ (P4, female, permanent physiotherapist)
4. Current management approaches	4.1. Lack of current tools used by physiotherapists 4.2. Referrals from other healthcare practitioners 4.3. Physiotherapists screening for pelvic health patients and rotations in pelvic health wards	4.1.1. ‘I use breathing, relaxation and pelvic floor strengthening exercises.’ (P1, male, permanent physiotherapist) 4.1.2. ‘Most doctors and other healthcare practitioners are unaware of physiotherapists’ role in pelvic health.’ (P1, male, permanent physiotherapist) 4.1.3. ‘We do not screen for pelvic patients due to understaffing.’ (P4, female, permanent physiotherapist)
5. Strategies to improve management of PFD	5.1. Physiotherapists to rotate in urology and gynaecology wards 5.2. Increase the availability of pelvic health training courses 5.3. Improve the physiotherapy undergraduate pelvic health curriculum to include practical skills 5.4. Educate healthcare practitioners and patients on the role of physiotherapists in pelvic health 5.5. Develop screening, referral, and treatment protocols	5.1.1. ‘There are not enough physiotherapists to rotate in all of the wards in the hospital.’ (P7, male, permanent physiotherapist) 5.1.2. ‘… it would be fair to provide more pelvic health courses that would accommodate the public sector.’ (P4, female, permanent physiotherapist) 5.1.3. ‘We need to improve the pelvic health content in undergraduate physiotherapy programmes.’ (P11, male, permanent physiotherapist) 5.1.4. ‘We need resources, pamphlets, and posters to educate patients and doctors on our role in pelvic health.’ (P3, male, community service physiotherapist) 5.1.5. ‘There are no available pelvic health treatment protocols.’ (P5, female, community service physiotherapist)

PFD, pelvic floor disorder.

### Theme 1: Undergraduate pelvic health training

The findings suggest that undergraduate physiotherapy programmes provide limited training in pelvic health, particularly in practical assessment and treatment skills. This lack of exposure may contribute to the insufficient confidence reported by participants. These findings are similar to Brandt (2025), who reported that pelvic health content in undergraduate programmes is often minimal and largely focused on antenatal and postnatal care. While pelvic floor exercises are commonly incorporated into antenatal and postnatal care, this statement reflects the participant’s perception that undergraduate training provided only limited exposure to pelvic health and did not equip them with detailed pelvic floor assessment or treatment skills. Similarly, Zamboni and Pleggenkuhle (2018) found that inadequate undergraduate training contributes to reduced confidence in managing pelvic health conditions. Given that physiotherapists are expected to manage conditions within obstetrics and gynaecology, this gap in training may negatively impact clinical competence.

Addressing this gap requires action at both institutional and professional levels. In the short term, physiotherapy departments could advocate for a minimum pelvic health competency framework to be embedded across all accredited undergraduate programmes – not as an incidental topic, but as a defined learning outcome spanning multiple year-levels. Shared clinical placements in gynaecology and urology wards, or inter-university pelvic health teaching days, could broaden practical exposure without requiring entirely new resources. In the long term, South African physiotherapy regulation bodies could work with training institutions to incorporate standardised pelvic health competencies into programme accreditation requirements, ensuring that all graduates enter the workforce with a functional foundation in this area.

### Theme 2: Physiotherapists’ barriers to managing pelvic floor disorders

Multiple barriers were identified, including religious, cultural, gender-related and systemic challenges. Religious and cultural beliefs may limit physiotherapists’ ability to treat certain patient groups, while gender-related discomfort highlights the sensitive nature of pelvic health practice. These barriers appear to be linked to limited experience and confidence, as suggested by Taak et al. (2024), who emphasised that pelvic health physiotherapy requires experienced practitioners.

Additionally, a lack of knowledge and confidence further restricts effective management, reinforcing the impact of insufficient training. Resource limitations, particularly the lack of private treatment areas, may compromise both patient comfort and quality of care. Limited referrals from other healthcare practitioners suggest poor interdisciplinary awareness, which has also been reported in previous South African and international studies (Koranteng et al. 2023; Reddy & Frantz 2013).

If other healthcare practitioners are unaware of physiotherapists’ role in pelvic health, the responsibility for changing that lies with the physiotherapy profession itself. Physiotherapists in these hospitals can take concrete steps: brief in-service presentations to medical and nursing staff on the management of urinary incontinence, pelvic organ prolapse and post-partum pelvic floor dysfunction represent a low-cost, effective starting point. One page referral guides outlining appropriate referral criteria can be displayed in doctors’ offices and nursing stations. Joint ward rounds in gynaecology and urology wards, where feasible, allow physiotherapists to demonstrate their role directly to colleagues. Regarding gender and cultural discomfort, departments could develop clear professional conduct frameworks for intimate assessments, including chaperone policies and standardised consent procedures, giving clinicians the professional scaffolding to manage sensitive encounters with greater confidence.

### Theme 3: Patients’ barriers to accessing physiotherapy

Patient-related barriers, including stigma, lack of awareness and cultural beliefs, were found to significantly impact access to care. The stigma associated with pelvic health conditions often prevents patients from seeking help, while misconceptions – such as urinary incontinence being a normal part of ageing – further delay intervention. These findings are similar to Nava et al. (2024) and McKay et al. (2019), who identified stigma and lack of knowledge as key barriers.

Additionally, financial constraints, such as transport costs and limited access to healthcare services, particularly in public-sector settings, are added barriers. These findings highlight the need for increased patient education and community outreach initiatives.

Stigma and unawareness diminish when the profession speaks openly and consistently. Community outreach visits to primary healthcare clinics, where most women in the district access care, offer a practical opportunity to deliver brief pelvic floor health education and normalise help-seeking. Simple, locally adapted, multilingual pamphlets and posters explaining what pelvic floor physiotherapy involves can be distributed through antenatal clinics, postnatal wards and via community health workers. Collaboration with community health workers, a cadre already trusted by patients in the district, may extend pelvic floor awareness far beyond what hospital-based physiotherapists alone can achieve.

### Theme 4: Current management approaches

The study found that physiotherapists primarily rely on basic treatment approaches, such as pelvic floor exercises, breathing and relaxation techniques. However, uncertainty regarding the appropriate application of these interventions suggests a gap in clinical knowledge. Furthermore, limited screening practices and lack of rotation in relevant wards reduce opportunities for early identification and management of PFDs.

The lack of awareness among healthcare practitioners regarding the role of physiotherapy further contributes to poor integration of services. This finding is supported by previous research indicating inadequate interdisciplinary collaboration in pelvic healthcare (Reddy & Frantz 2013).

Where dedicated ward rotations are not feasible due to staffing constraints, a brief structured pelvic floor screening tool embedded into routine physiotherapy assessments of postnatal, spinal cord injury or urology patients could identify cases without requiring additional staff time. Group-based pelvic floor exercise classes, widely used in higher-resource settings (Slade et al. 2020), allow one physiotherapist to deliver evidence-based intervention to multiple patients simultaneously, improving reach without proportionally increasing workload. The development of locally adapted, context-specific assessment and treatment protocols drawing on existing international guidelines (Fernández-Pérez et al. 2023; Grimes & Stratton 2023) would reduce dependence on individual clinical knowledge and create a reproducible standard of care that all physiotherapists in the district could follow.

### Theme 5: Strategies to improve management of pelvic floor disorders

Participants proposed several strategies to improve pelvic health management. These include increasing access to training opportunities, improving undergraduate curricula and developing standardised assessment and treatment protocols. Enhancing interdisciplinary education and raising awareness among healthcare practitioners may improve referral pathways. Additionally, improving staffing and enabling physiotherapists to rotate through urology and gynaecology wards may facilitate better service delivery. Patient education initiatives were also highlighted as essential to reduce stigma and improve healthcare-seeking behaviour.

While systemic changes such as increased staffing and infrastructure investment remain important long-term goals, the profession need not wait for these to begin improving care. Peer learning circles within districts offer an immediately feasible and cost-effective route to spreading expertise. Professional associations could negotiate subsidised continuing professional development (CPD) for public sector physiotherapists, while digital platforms and recorded webinars can make specialist content accessible in rural and under-resourced settings (Alaniz et al. 2020; Boissonnault et al. 2020; Brandt 2025). Taken together, the strategies proposed by participants in this study, combined with models already operating in comparable contexts, suggest that meaningful improvement in pelvic health physiotherapy management in the uThukela district is achievable and that the profession has both the opportunity and the responsibility to lead it.

### Study limitations

This study was conducted in three district hospitals within a single health district, which may limit the transferability of the findings to other settings. The relatively small sample size, inherent to qualitative research, may not capture the full range of physiotherapists’ experiences across public healthcare contexts. Data were collected via telephonic interviews, which may have limited the depth of interaction and the ability to observe non-verbal cues.

### Recommendations

Further research should investigate and develop suitable physiotherapy protocols for pelvic health assessment and treatment to facilitate effective pelvic health management in the uThukela district.

## Conclusion

This study is unique in that it examined the experiences of physiotherapists in managing PFDs in the uThukela district. The study highlighted barriers that prevent physiotherapists from managing pelvic health conditions, such as physiotherapy undergraduate programmes not covering pelvic health management skills in detail, a lack of available pelvic health postgraduate training in the public sector, a lack of pelvic health referrals to physiotherapy from other healthcare practitioners, a lack of resources in the public sector and physiotherapy understaffing. The study further identified barriers that patients experience that prevent them from receiving pelvic physiotherapy management, such as the stigma attached to pelvic health and the lack of awareness of the role of physiotherapy in pelvic health. The study demonstrated the need for undergraduate physiotherapy programmes to improve their pelvic health curricula, to educate other healthcare practitioners on the role of physiotherapists in pelvic health, to encourage pelvic health promotion to patients, to increase the availability of pelvic health physiotherapy training in the public sector, and to improve hospital resources and staffing.
